# Hormonal and metabolic responses across phases of combined oral contraceptive use and menstrual cycle in young elite female athletes

**DOI:** 10.1007/s00421-025-05745-x

**Published:** 2025-03-09

**Authors:** Katia Collomp, Agnès Olivier, Caroline Teulier, Juliette Bonnigal, Nathalie Crépin, Corinne Buisson, Magnus Ericsson, Emmanuelle Duron, Eric Favory, Mathieu Zimmermann, Virgile Amiot, Carole Castanier

**Affiliations:** 1 CIAMS, Université d’Orléans, Pôle STAPS, Orléans, France; 2 CIAMS, Université Paris-Saclay, Orsay, France; 3 Fédération SAPREM, Université d’Orléans, Orléans, France; 4 LADF, Université Paris-Saclay, Orsay, France; 5 IFCE, Cadre Noir, Saint-Hilaire-Saint-Florent, Saumur France; 6 Hôpital Brousse, AP-HP, Equipe INSERM MOODS-CESP, Villejuif, Kremlin-Bicêtre France; 7 Médecine du Sport, CHU Orléans, Orléans, France

**Keywords:** Ethinylestradiol/levonorgestrel, Sex-hormone status, Highly trained, TSH, Aldosterone, 25(OH)D

## Abstract

**Purpose:**

Despite the significant number of female athletes using combined oral contraceptives (COCs), there is scant literature on their hormonal and metabolic effects across different phases.

**Methods:**

In order to contribute to a wider knowledge of COC-action mechanisms involved in athletes’ performance and health, we therefore examined the effects of low-dose monophasic COC (ethinylestradiol/levonorgestrel) intake on sex hormones (estradiol, progesterone, sex hormone binding protein (SHBG)) as well as on a large number of pituitary (LH, TSH, prolactin) and peripheral (triiodothyronine, cortisol, DHEA, DHEA-S, aldosterone, osteocalcin, 25(OH)D) basal hormone levels in nine young elite female athletes, across COC administration (first and second half of active hormone intake, washout phases), compared to eleven female athletes without hormonal contraception across their normal menstrual cycle (NMC, i.e., early follicular, end follicular/peri-ovulatory, mid-luteal phases).

**Results:**

COC vs. NMC increased SHBG (p < 0.01), TSH, cortisol and 25(OH)D (p < 0.05), and decreased DHEA and DHEA-S (p < 0.05) concentrations. Across COC and NMC phases, higher estradiol and aldosterone concentrations (p < 0.05) were observed during the washout and mid-luteal phases, respectively.

**Conclusion:**

In highly trained female athletes, COC vs. NMC induced several hormonal alterations, irrespective of the phases, leading to potential ergogenic and clinical repercussions that merit clarification. In NMC athletes, the impact of endogenous sex hormone fluctuations on the parameters studied appeared limited, perhaps mitigated by intense physical training, with only aldosterone change. Given the high prevalence of vitamin D insufficiency, it seems warranted to monitor this parameter, not yet routinely considered in female athletes, taking into account COC intake.

**Trial registration** : ID-RCB:2020-A02965-34, France

**Graphical Abstract:**

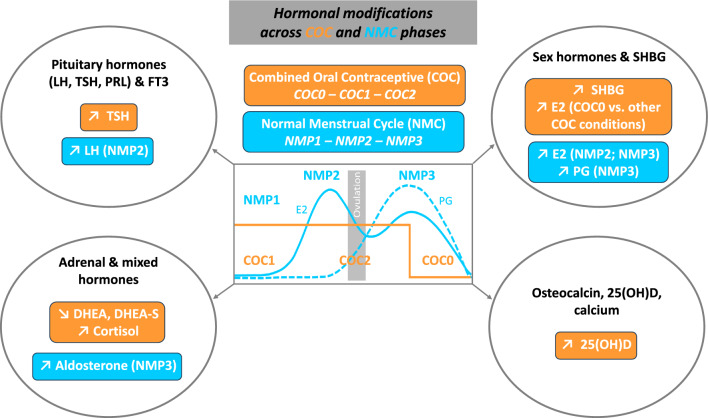

## Introduction

Between 20 and 70% of elite female athletes use hormonal contraception (Larsen et al. 2020; Martin et al. 2018; Oxfeldt et al. 2020), with a large majority opting for 2nd-generation combined oral contraceptives (COCs). These COCs, whether mono-, bi- or triphasic, contain synthetic estrogens and progestins, typically ethinylestradiol (EE) as the estrogen at concentrations between 20 and 50 μg per pill and levonorgestrel (LVN) as the progestin at concentrations of 50 to 150 μg (Castanier et al. 2021). They are administered for 21 days followed by a 7 day break (i.e., washout phase), with or without inactive non-hormonal pills, to mimic the menstrual cycle but without the peaks of estradiol (E2) and progesterone (PG), thus stabilising hormone levels through inhibition of the hypothalamo-pituitary-gonadal (HPG) axis and suppression of gonadotropin secretion produced by the pituitary gland, follicle-stimulating hormone (FSH) and luteinizing hormone (LH).

While COCs are known to significantly decrease E2 and PG, free and total blood testosterone, as well as dehydroepiandrosterone (DHEA) and dehydroepiandrosterone-sulphate (DHEA-S), with a parallel increase in sex hormone-binding protein (SHBG) concentrations in healthy women (Coelingh Bennink et al. 2017; Enea et al. 2009; Knutsson et al. 2023), the literature on a female athlete population or on the other COC hormonal effects is sparse and lacking consensus. Indeed, few studies, most of which were carried out on non-physically trained women and/or with higher COC doses, reported during the active hormonal pill phase an increase or not in prolactin (PRL), thyroid-stimulating hormone (TSH), free triiodothyronine (FT3), cortisol (COR), aldosterone (ALD), osteocalcin (OST) and calcidiol (25(OH)D) (Aden et al. 1998; Kuhl et al. 1985a, 1985b; Özcan et al. 2023; Raps et al. 2014; Sawin et al. 1978; Weeke and Hansen 1975; Wiegratz et al. 2003). As these hormones directly or indirectly regulate metabolism, COC-induced changes may play a role in athletes' performance and health. Moreover, even fewer studies have explored potential modifications in hormonal and metabolic status during the COC washout phase (Aden et al. 1998; Ihalainen et al. 2021; Kuhl et al. 1985a; Martin et al. 2021; Rechichi et al. 2008; Weeke and Hansen 1975) and across the intake period, despite LVN concentrations only reaching a steady state in the second half of each active hormone intake treatment cycle (Kuhnz et al. 1992).

In order to contribute to a wider knowledge of COC-action mechanisms in highly trained female athletes, we therefore aimed to examine the effects of low-dose monophasic COC (i.e. EE/LVN) intake on various pituitary and peripheral hormone secretions and metabolic responses in young elite female athletes, taking into account the phase of COC administration (first and second half of active hormone intake, and washout phases), compared to female athletes with a normal menstrual cycle (NMC), during the early follicular phase (low E2), end follicular/peri-ovulatory phase (high E2 & low PG) and mid-luteal phase (high E2 & high PG). We hypothesised that COC vs. NMC will induce several marked changes in these pituitary and peripheral hormones in an elite female population, with modulation across the different phases of active/inactive pill-taking. In parallel, we will assess in NMC athletes the impact of endogenous sex hormone fluctuations across the menstrual cycle on the parameters studied.

## Methods

### Participants

Twenty elite female athletes (judokas and riders), recruited from centers and technical platforms hosting top-level athletes, volunteered for the study (age: 19.6 ± 0.4 years; height: 164.4 ± 2.5 cm; weight: 62.0 ± 2.6 kg; BMI: 22.9 ± 0.5 kg/m2; % fat mass: 19.8 ± 2.0). After being informed of the study’s objectives and risks, participants signed a written consent form. All procedures performed in the study were approved by the ethics committee (ID-RCB:2020-A02965-34) and were in accordance with the Declaration of Helsinki.

Prior to the study, subjects were screened with a medical history and physical examination. Inclusion criteria required participants to be highly physically active (6 to 11 training sessions per week, 3 to 6 h per day, for at least 3 years). Exclusion criteria included cardiovascular, liver, biliary or renal disease; hyperlipidaemia; uncontrolled high blood pressure; endocrinological disorder; oligomenorrhoea or amenorrhoea; pregnancy; history of thromboembolic disorder. Subjects were divided into 2 groups: Group 1 (9 subjects) had used monophasic COC: 20 μg/100 μg (7); 30 μg/150 μg (2) of ethinylestradiol and levonorgestrel per pill, respectively, for at least six months, with pills during the washout phase for all participants; Group 2 (11 subjects) had NMC, without hormonal contraception for at least 1 year. Subjects reported regular menstrual cycle between 25 and 29 days during the last three cycles.

### Experimental procedure

Each participant visited the laboratory 3 times after the inclusion visit, at 5 week intervals, in order to systematically shift the subjects' cycle phase and reach the 3 target statuses, in the winter and spring seasons, at mid-latitude. For the COC group, visits were scheduled during the first (Day3–Day10, i.e. COC1) and second (Day11–Day18, i.e. COC2) periods of pill intake as well as during the washout without hormone intake (W2–W7, i.e. COC0). For the NMC group, visits occurred during the early follicular phase (Day1–Day7, i.e. NMP1) with Day1 being the first day of bleeding, the end follicular/peri-ovulatory phase (Day8–Day14, i.e. NMP2) and during the mid-luteal phase (Day18–Day24, i.e. NMP3). Ovulation was detected through PG measurement in all NMC participants, and menstrual phases were retrospectively confirmed by analyzing serum sex female hormones.

### Blood collection and analysis

Blood samples were collected in the morning (7:00–8:00 a.m.) after an overnight fast. Participants were asked to maintain their normal dietary intake during the experimental period, and to refrain from consuming caffeine or alcohol and from making strenuous efforts for at least 24 h prior to the trial sessions. Venous blood samples were collected from an antecubital vein using standard procedures, and the blood was transferred into serum, EDTA and heparin tubes. The serum and plasma were obtained by centrifuging the tubes at 3500 rpm for 10 min before being stored at −20 °C for later analysis. Vitamin D (25(OH)D) was determined by electrochemiluminescence, and calcium by a classical photometric test. The concentrations of E2, PG, SHBG, LH, PRL, TSH, FT3, DHEA, DHEA-S, COR, ALD and OST were determined by ELISA (kits from DRG Diagnostic®, Germany). The free estradiol index (FEI) was determined by [E2/SHBG]. The analytical sensitivities for the assays were: Vitamin D: 3.0 ng/ml; calcium: 8 mg/L; E2: 10.6 pg/ml; PG: 0.045 ng/ml; SHBG: 0.41nmol/L; LH: 1.27 mIU/ml; PRL: 0.35 ng/ml; TSH: 0.06 mIU/ml; FT3: 0.38 pg/ml; DHEA-S: 0.04 mg/L; DHEA: 0.63 ng/ml, COR: 1.3 ng/ml, ALD: 10.65 pg/ml and OST: 0.31 ng/ml, respectively. Assays were made in duplicate, and coefficients of variation for all parameters were always < 10%.

### Statistical analyses

Results are presented as mean values ± standard error of the mean (SEM). After evaluating the normality of the samples, differences in blood parameters between the trials were analyzed with a two-way (treatment and phase) analysis of variance (ANOVA) with repeated measurements. A post hoc Fisher test was performed to locate the differences, in the event of an ANOVA revealing a significant main effect. Size of treatment was estimated with Cohen’s d scores and 95% CI for d. Correlations were calculated using Pearson’s product moment correlation test. The null hypothesis was rejected at p < 0.05.

## Results

### Sex hormones, SHBG and FEI (Figs. 1 and 2)

**Fig. 1:**
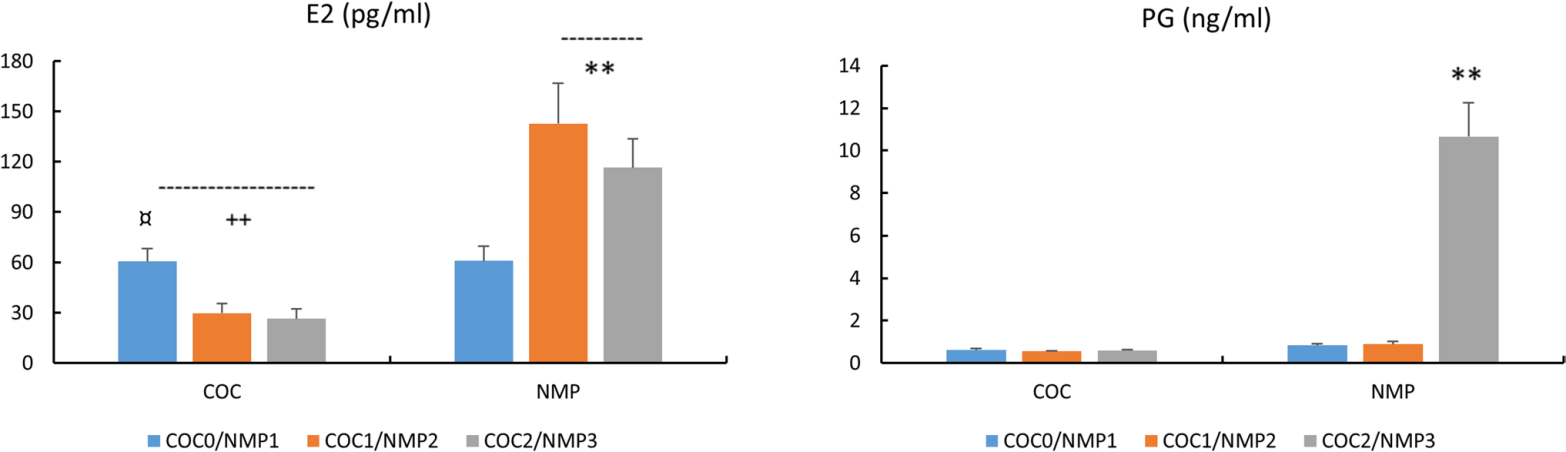
Concentrations of E2 (pg/ml) and PG (ng/ml) in COC and NMC subjects. For COC: COC0 (washout); COC1 (1st half of active pill); COC2 (2nd half of active pill); For NMC: NMP1 (early follicular); NMP2 (end follicular/peri-ovulatory); NMP3 (mid-luteal)^+^,p < 0.05,^++^, p < 0.01, difference COC vs. NMC *, p < 0.05, **, p < 0.01, difference across NMC phases, ¤p < 0.05, difference across COC phases

**Fig. 2:**
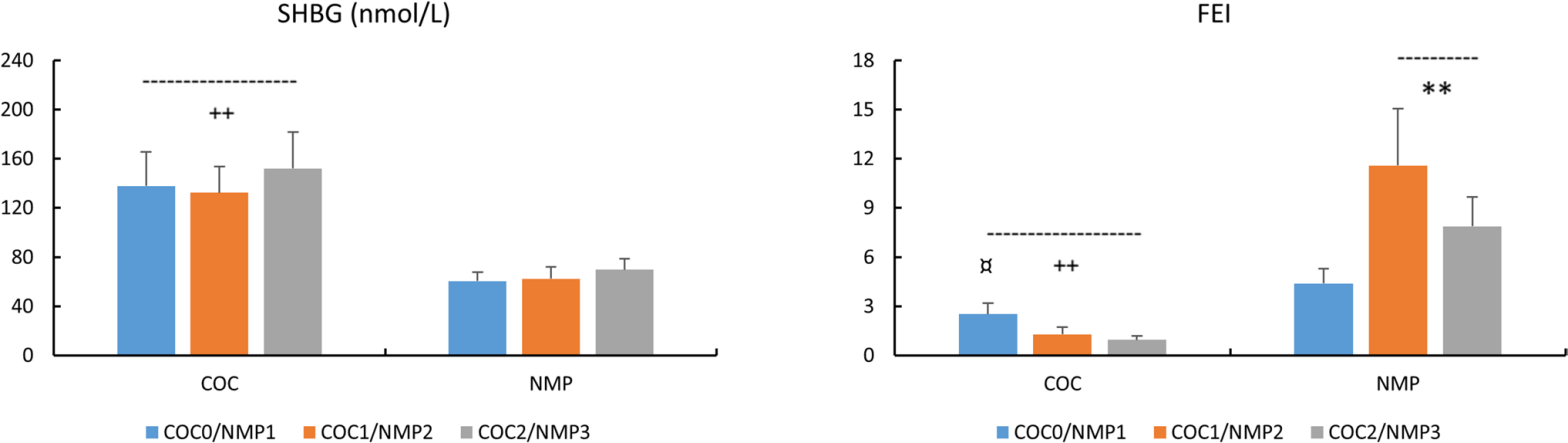
Concentrations of SHBG (nmol/L) and free estradiol index (FEI) in COC and NMC subjects For COC: COC0 (washout); COC1 (1st half of active pill); COC2 (2nd half of active pill); For NMC: NMP1 (early follicular); NMP2 (end follicular/peri-ovulatory); NMP3 (mid-luteal)^+^, p < 0.05, ^++^, p < 0.01, difference COC vs. NMC *, p < 0.05, **, p < 0.01, difference across NMC phases, ¤p < 0.05, difference across COC phases

E2 and PG concentrations were lower in COC vs. NMC (p < 0.01, Cohen’s d: −1.57, 95% CI for d: −2.15,−0.99; p < 0.01, Cohen’s d: −2.34, 95% CI for d: −3.00,−1.69, respectively). There was also a treatment effect for SHBG and FEI, with higher values in COC vs. NMC for SHBG (p < 0.01, Cohen’s d: 1.35, 95% CI for d: 0.79,1.91), and lower values for FEI (p < 0.01, Cohen’s d: −1.23, 95% CI for d: −1.79,−0.78). In COC subjects, higher E2 was obtained in COC0 vs. COC1 and COC2 (p < 0.05). In NMC subjects, higher E2 was obtained in NMP2 and NMP3 vs. NMP1, and higher PG in NMP3 vs. NMP2 and NMP1 (p < 0.01). No difference in E2 and FEI between COC0 and NMP1was found.

### Pituitary hormones and FT3 (Fig. 3)

**Fig. 3:**
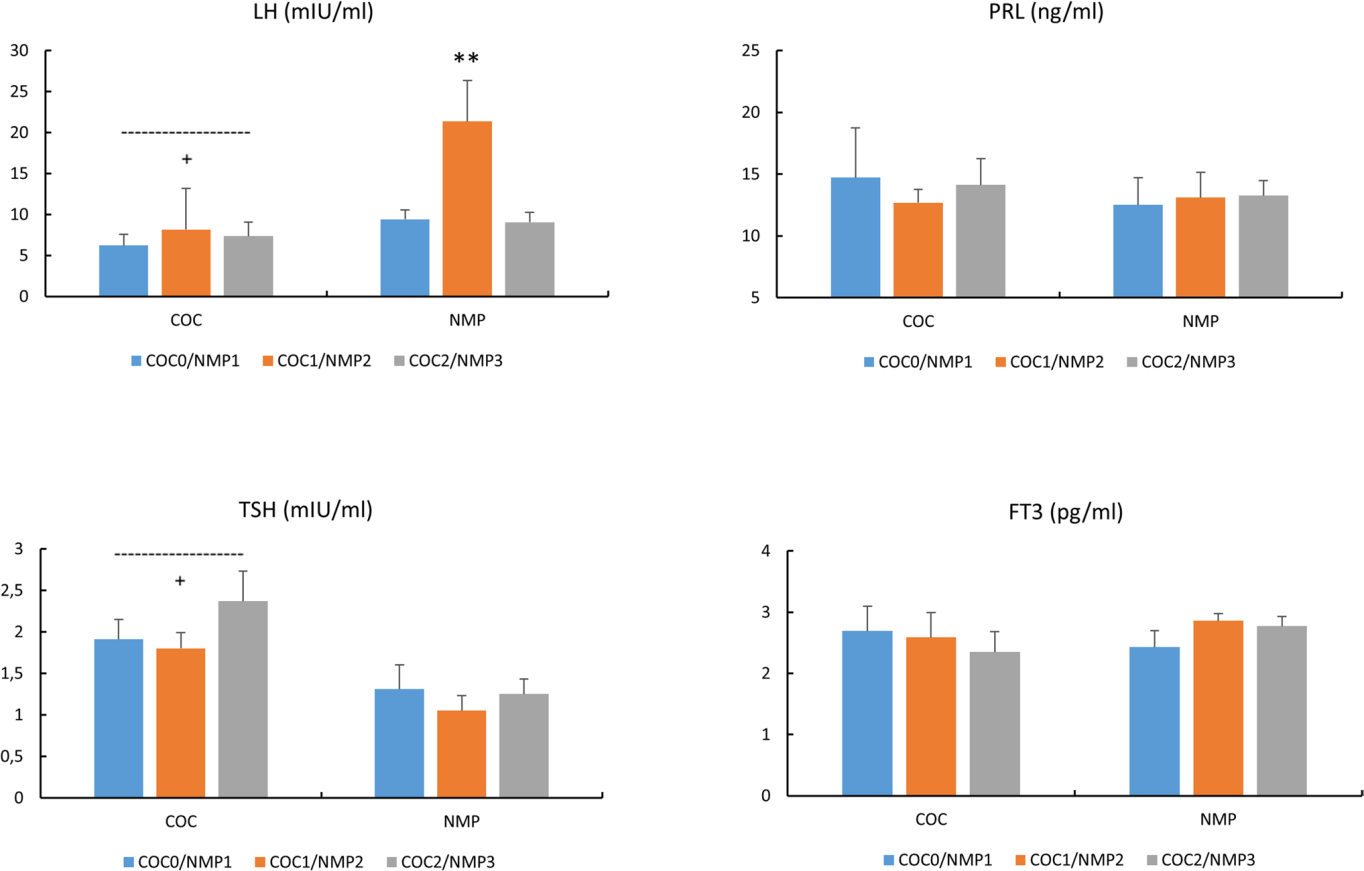
Concentrations of LH (mIU/ml), PRL (ng/ml), TSH (mIU/ml) and FT3 (pg/ml) in COC and NMC subjects For COC: COC0 (washout); COC1 (1st half of active pill); COC2 (2nd half of active pill); For NMC: NMP1 (early follicular); NMP2 (end follicular/peri-ovulatory); NMP3 (mid-luteal)^+^, p < 0.05, difference COC vs. NMC *, p < 0.05, **, p < 0.01, difference across NMC phases

There were decreased LH and increased TSH (p < 0.05, Cohen’s d:—0.91, 95% CI for d: −1.44,−0.38; Cohen’s d: 1.09, 95% CI for d: 0.55,1.64 respectively) concentrations in COC vs. NMC. No treatment effect occurred for either PRL or FT3 concentrations. There was an increased LH in NMP2 vs. other conditions (p < 0.01).

### Adrenal and mixed hormones (Fig. 4)

**Fig. 4:**
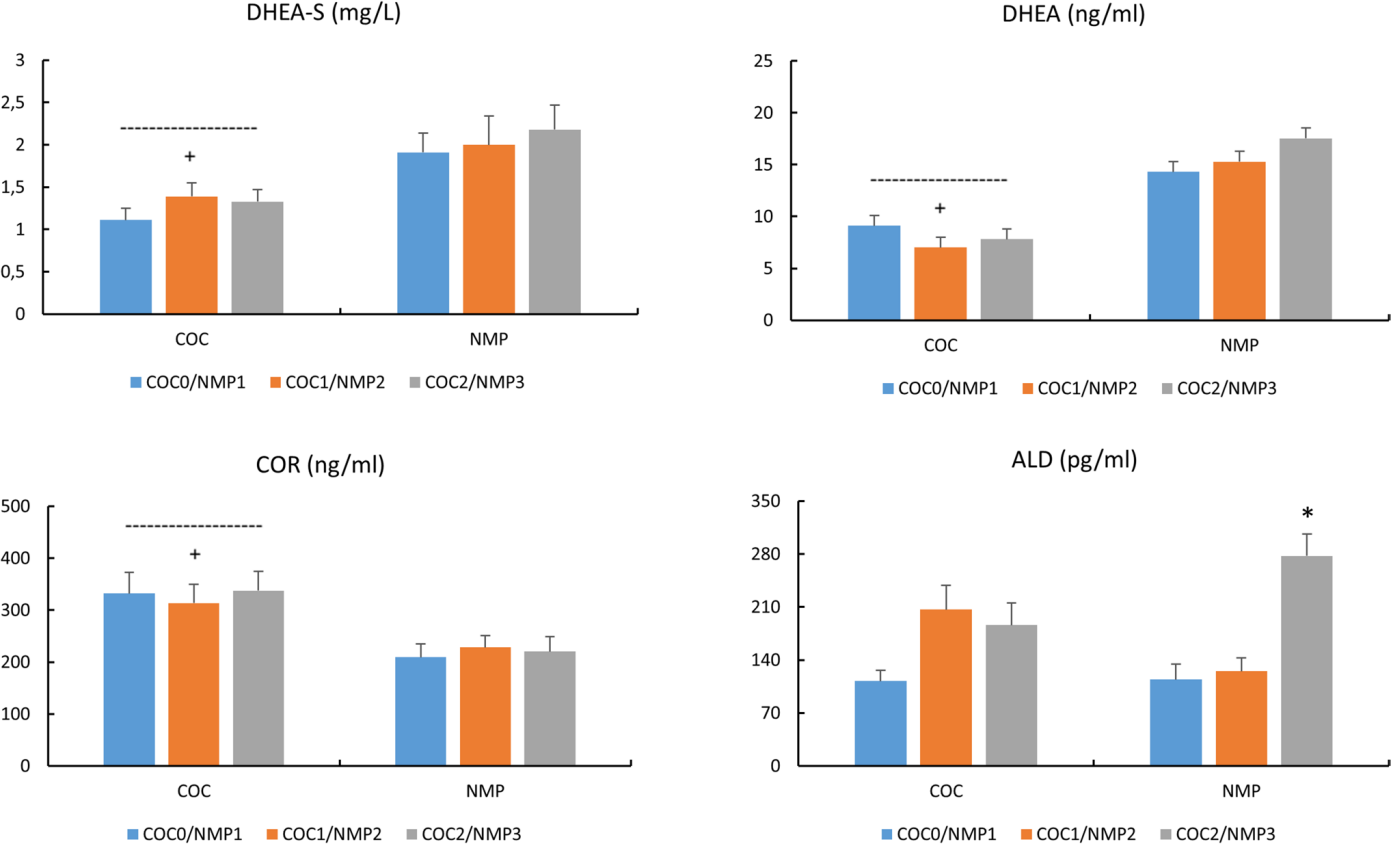
Concentrations of DHEA-S (mg/L), DHEA (ng/ml), COR (ng/ml) and ALD (pg/ml) in COC and NMC subjects For COC: COC0 (washout); COC1 (1st half of active pill); COC2 (2nd half of active pill); For NMC: NMP1 (early follicular); NMP2 (end follicular/peri-ovulatory); NMP3 (mid-luteal)^+^, p < 0.05, difference COC vs. NMC *, p < 0.05, difference across NMC phases

DHEA (p < 0.05, Cohen’s d: −1.44, 95% CI for d: −2.02,−0.87) and DHEA-S (p < 0.05, Cohen’s d: −1.04, 95% CI for d: −1.59,−0.51) concentrations were lower and COR levels higher in COC vs. NMC (p < 0.05, Cohen’s d: 1.06, 95% CI for d: 0.55,1.53). There was a significant increase in ALD in NMP3 vs. the other conditions (p < 0.05).

### Osteocalcin, vitamin D, and calcium (Fig. 5)

**Fig. 5:**
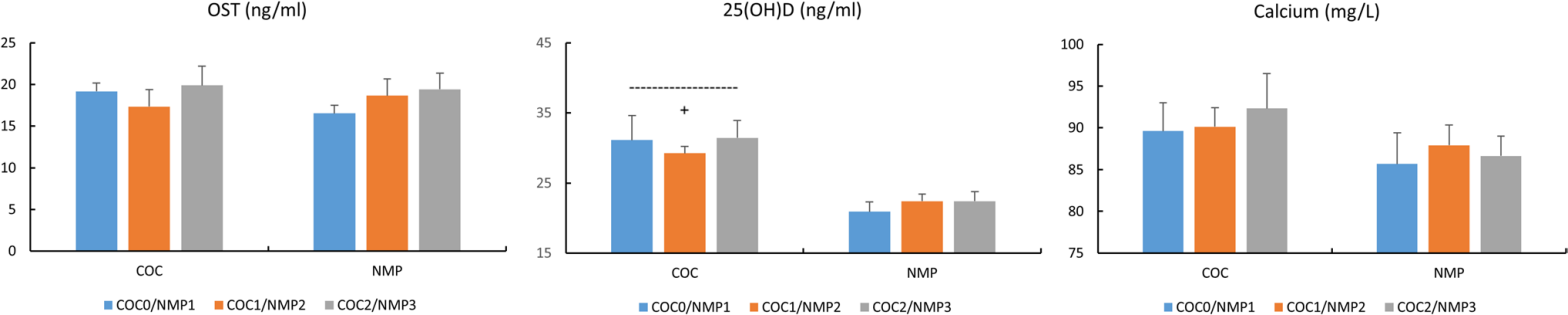
Concentrations of OST (ng/ml), 25(OH)D (ng/ml) and calcium (mg/L) in COC and NMC subjects For COC: COC0 (washout); COC1 (1st half of active pill); COC2 (2nd half of active pill); For NMC: NMP1 (early follicular); NMP2 (end follicular/peri-ovulatory); NMP3 (mid-luteal) ^+^, p < 0.05, difference COC vs. NMC

No change in COC vs. NMC was found for osteocalcin or calcium, whereas a treatment difference was found for 25(OH)D, with higher values in COC vs. NMC (p < 0.05, Cohen’s d: 1.27, 95% CI for d: 0.71,1.82). No change in either phase was observed for these parameters.

### Correlation

Considering all subjects, there was a significant correlation (p < 0.05) between E2 and LH (r = 0.59), E2 and DHEA (r = 0.62), DHEA and DHEA-S (r = 0.59), SHBG and cortisol (r = 0.56), SHBG and TSH (r = 0.46), SHBG and 25(OH)D (r = 0.46). In NMC, there were significant correlations (p < 0.05) between PG and ALD (r = 0.56) as well as between FT3 and 25(OH)D (r = 0.47).

## Discussion

Given the scarcity of studies carried out on female athletes, we examined in this population the impact of COC (EE/LVN), across the different phases of active/inactive pill-taking, on various pituitary and peripheral hormones that play an important role in athlete performance and health. In parallel, we assessed in NMC athletes the effects of endogenous sex hormone fluctuations across the menstrual cycle on the parameters studied. Intake of COC vs. NMC, irrespective of the phase, increased TSH, SHBG, cortisol and 25(OH)D, and decreased DHEA and DHEA-S concentrations. Few parameters were influenced by the COC or NMC phases, with only E2 and aldosterone concentrations being higher during the washout and mid-luteal phases, respectively.

As anticipated (Castanier et al. 2021; Martin et al. 2018; Oxfeldt et al. 2020), in NMC subjects, we observed higher concentrations of LH during NMP2, with significantly elevated estradiol concentrations during NMP2 and NMP3 compared to NMP1. Progesterone, significantly higher during NMP3, was utilized to verify ovulation and thus the normality of the menstrual cycle in our NMC subjects. COCs logically inhibited ovulation, resulting in no LH peak nor PG increase, with significantly lower E2 concentrations in COC versus NMC subjects, except during the washout phase. Interestingly, there was a significant increase in E2 secretion during this hormone-free period, compared to the period of hormone intake, with similar values versus the early follicular phase. There is no consensus on this increase, or lack thereof, in E2 during COC0. This may be explained by the type of COC used and/or the timing of sampling. Indeed, our subjects were tested after an average washout of four days, following low doses of EE/LNG, unlike studies conducted on female athletes at the start of the inactive pill or with unspecified progestin nature (Ihalainen et al. 2021). In line with the literature (Coelingh Bennink et al. 2017; Knutsson et al. 2023; Özcan et al. 2023), we observed an increase in SHBG concentrations by more than 100% in our study with COCs, irrespective of the phase of COC administration. However, free estradiol index (E2/SHBG), considered to reflect estradiol activity (Gilberg et al. 2002), was not significantly different during the washout phase compared to the early follicular phase, in contrast to the first and second half of active hormone intake, where it was strongly decreased compared to NMC subjects. This suggests a relative increase in endogenous estrogenic activity during the COC-washout vs. COC-active hormone phases, which could partially compensate for the drop in exogenous hormone levels.

Prolactin and thyroid hormones each have pleotropic and independent roles allowing the body to accommodate to physical activity and exercise but they also share an interrelation in their responses, since hypothalamic thyrotropin-releasing hormone (TRH) release stimulates the release of TSH and thus the thyroids as well as the release of PRL, estrogen serving as an interconnective regulatory link by stimulating the release of both the thyroids and prolactin (Hackney and Saeidi 2019). Despite the variations of endogenous/exogenous estrogen concentrations across NMC and COC phases, no change in PRL levels was observed in the present study, either as a function of the menstrual cycle or hormonal contraception, and similarly, TSH values remained consistent irrespective of the menstrual cycle period. These results align with previous studies reporting little or no effect of the endogenous hormonal status in women (Aden et al. 1998; Kuhl et al. 1985a; Sawin et al. 1978; Weeke and Hansen 1975). However, we found increased TSH but not FT3 concentrations in our COC subjects, maintaining this increase during all COC periods. Higher TSH concentrations with COCs were initially described with concomitant higher total and free T3 and T4 concentrations (Kuhl et al. 1985b; Weeke and Hansen 1975), but most recent studies have reported an increase in TSH but not in free thyroid hormones, due to the parallel increase in thyroxine-binding globulin (TBG) protein (Özcan et al. 2023; Raps et al. 2014; Wiegratz et al. 2003). It may therefore be suggested that the discrepancy may be related to the lower doses of synthetic estrogens and progestins used in COCs today, limiting their thyroid impact.

With COC administration, we found a significant decrease in both DHEA and DHEA-S compared to NMC. This finding agreed with experiments performed on sedentary or recreationally active female subjects, with COCs including different progestins (Coelingh Bennink et al. 2017; Knutsson et al. 2023). COCs lower androgen status may drive mood and sexual function alterations in women although studies of these effects have yielded conflicting results (Burrows et al. 2012; Coelingh Bennink et al. 2017; Oinonen and Mazmanian 2002). Similarly, the higher total serum COR obtained here in the COC versus the NMC group was consistent with most previous works (Özcan et al. 2023). Unfortunately, we did not analyze cortisol-binding protein (CBP), but studies showed that COCs stimulate the liver production of binding proteins, including CBP, with, consequently, a probable lack of change in free cortisol concentrations and/or effects (Özcan et al. 2023; Vibarel-Rebot et al. 2015). Regarding NMC phases, according to the literature, we found no modification in the androgenic adrenal/gonadal DHEA and DHEA-S concentrations nor in COR throughout the menstrual cycle (Kanaley et al. 1992; Kuhl et al. 1985a; Knutsson et al. 2023; Vibarel-Rebot et al. 2015), whereas aldosterone levels were significantly increased in NMP3 compared with other conditions. There is very little data on aldosterone variations during the menstrual cycle, but one study (De Souza et al. 1989) previously noticed an increase during the mid-luteal phase. It may be hypothesized that this increase, whose involvement during endurance exercise needs to be determined, was mediated by the increase in its PG precursor, in view of the significant correlation obtained (r = 0.56).

Estrogens are considered as the “key regulator” of bone metabolism in women (Khosla and Monroe 2018) and changes in vitamin D and osteocalcin concentrations have been previously reported either across the menstrual cycle or with COC intake in healthy women or female athletes (Harmon et al. 2016, 2020; Jürimäe et al. 2011). Vitamin D is a fat-soluble steroid pro-hormone that occurs in two forms, vitamin D2 and D3, which may be obtained from sunlight exposure of the skin as well as from the diet. Both vitamin D2 and D3 undergo hydroxylation in the liver, where they are converted into 25(OH)D, further hydroxylated in the kidney to form 1,25-hydroxyvitamin D (calcitriol), a biologically active metabolite which then binds to vitamin D receptors (VDBP) at target tissues (Farrokhyar et al. 2015; Harju et al. 2022; Sist et al. 2023). Despite its well-recognized importance for both skeletal and non-skeletal systems, including cardiovascular, immune, inflammatory and muscle functions, a large proportion of the sporting population is deficient in vitamin D, with the risk increasing significantly in high latitudes, as well as in winter and spring, although specific prevalence in female athletes remains unknown (Farrokhyar et al. 2015; Harju et al. 2022; Sist et al. 2023). In our NMC group, 25(OH)D concentrations were below the recommended value of 30 ng/ml in most of our subjects (9/11), with two of them even showing vitamin D deficiency (< 20 ng/ml). These percentages (80%, 45% and 20% below 30, 25 and 20 ng/ml, respectively) appeared greater than those observed in sedentary or moderately active women (Harmon et al. 2020) and may reflect the increased needs resulting from intensive physical training. The same previous study only reported minimal change in calciotropic hormones during the menstrual cycle (Harmon et al. 2020) but, in women with 25(OH)D values below 30 ng/ml, lower mean E2 across the menstrual cycle. Partially in line with this study, we found similar 25(OH)D values across the menstrual cycle, probably because of its long half-life, but without any correlation with E2 levels. Interestingly, we observed in parallel relatively low calcium concentrations in our population and significant correlation between 25(OH)D and FT3 concentrations. There was surprisingly little information in female athletes (Nikolaidis et al. 2003) but it may be suggested that low blood calcium and FT3 may be partially resulted from low 25(OH)D values. Lastly, we found similar osteocalcin levels across the menstrual cycle or between our groups of subjects. This finding was partially in accordance with previous studies (Jürimäe et al. 2011; Martin et al. 2021) reporting no change across NMC but lower osteocalcin with COC administration (i.e., 20 mg of EE and 75 mg of gestodene) compared to NMC (Jürimäe et al. 2011). One hypothesis to explain the discrepancy with our COC data is the use of different progestins, whose specific impact on osteocalcin remains to be confirmed. However, our COC subjects had significantly higher 25(OH)D versus NMC subjects, with only one subject with vitamin D deficiency. This increase in 25(OH)D by COC intake was previously reported (Harmon et al. 2016) but as COC induced in parallel a 30% VDPB increase (Özcan et al. 2023), vitamin D status may be overestimated in COC subjects and the biological pathways involved remain to be determined (Harmon et al. 2016).

## Conclusion

In highly trained female athletes, low-dose monophasic COC induced, compared to NMC, several hormonal alterations, irrespective of the active/inactive hormone intake phases, leading to potential ergogenic and clinical repercussions that merit clarification. In NMC female athletes, the impact of endogenous sex hormone fluctuations on the parameters studied appears to be very limited, perhaps mitigated by intense physical training, with only a change in aldosterone concentrations. Finally, given the high prevalence of vitamin D insufficiency, it seems warranted to monitor this parameter, not yet routinely considered in female athletes, taking into account COC intake as well as training, competition, season and environmental conditions (Farrokhyar et al. 2015; Kraus et al. 2020).

## Data Availability

The data that support the findings are available from the corresponding author upon reasonable request.

## Abbreviations

ALD: Aldosterone
ANOVA: Analyse of variance
CBP: Cortisol-binding protein
COC: Combined oral contraceptive
COC0: Washout period (inactive hormone intake)
COC1: First half of active hormone intake
COC2: Second half of active hormone intake
COR: Cortisol
DHEA: Dehydroepiandrosterone
DHEA-S: Dehydroepiandrosterone-sulphate
EE: Ethinylestradiol
E2: Estradiol
FEI: Free estradiol index
FSH: Follicle-stimulating hormone
FT3: Free triiodothyronine
HPG: Hypothalamo-pituitary–gonadal
LH: Luteinizing hormone
LVN: Levonorgestrel
NMC: Normal menstrual cycle
NMP1: Early follicular phase
NMP2: End follicular/peri-ovulatory phase
NMP3: Mid-luteal phase
OST: Osteocalcin
PG: Progesterone
PRL: Prolactin
SEM: Standard error of the mean
SHBG: Sex hormone binding protein
TSH: Thyroid-stimulating hormone
25(OH) D: Calcidiol (25-hydroxyvitamin D)
